# The SARAH trial: more evidence on the role of neurohormonal blockers in prevention of anthracycline-induced cardiotoxicity

**DOI:** 10.1007/s10741-025-10495-1

**Published:** 2025-02-17

**Authors:** Albulena Mecinaj, Victoria Vinje-Jakobsen, Doan T. M. Ngo, Aaron L. Sverdlov, Peder L. Myhre

**Affiliations:** 1 Department of Cardiology, Akershus University Hospital, Lørenskog, Norway; 2 K.G. Jebsen Center for Cardiac Biomarkers, Institute of Clinical Medicine, University of Oslo, Oslo, Norway; 3 Newcastle Centre of Excellence in Cardio-Oncology, Hunter Medical Research Institute, Hunter New England Local Health District, University of Newcastle and Calvary Mater Newcastle, New Lambton Heights, Newcastle, Australia; 4 College of Health Medicine and Wellbeing, University of Newcastle, Callaghan, Australia; 5 Cardiovascular Department, John Hunter Hospital, New Lambton Heights, Australia; 6 Department of Cardiology, Oslo University Hospital Rikshospitalet, Oslo, Norway

**Keywords:** Cardio oncology, Anthracycline, Cardiac function, Sacubitril/valsartan, Angiotensin receptor neprilysin inhibitor

## Abstract

This mini-review highlights the results of the SARAH trial, which evaluated the efficacy of sacubitril/valsartan in preventing subclinical cardiac dysfunction in patients undergoing anthracycline-based chemotherapy. The trial demonstrated a significant reduction in GLS decline in the treatment group compared to placebo. The findings are discussed in context with other trials investigating neurohormonal blockade for the prevention of cancer therapy-related cardiac dysfunction.

Cancer therapy-related cardiac dysfunction (CTRCD) remains a significant concern in patients undergoing cancer therapy. This can be further exacerbated by HER2-targeted therapies, which are administered following the anthracyclines (or other chemotherapies) for patients with HER2-positive breast cancer. Cardiotoxic effects range from asymptomatic rise in cardiac enzymes and left ventricular ejection fraction (LVEF) decline to overt heart failure. In addition to possibly interrupting lifesaving cancer therapy, even subclinical adverse cardiac effects may increase the risk of worse long-term cardiovascular outcomes. With cardiovascular disease remaining a leading cause of morbidity and mortality in this population, amelioration of cardiotoxicity represents a major area of unmet clinical need.

The key cardiotoxic mechanisms of anthracycline are believed to involve alterations of cell death pathways, including the inhibition of topoisomerase II and ferroptosis, and the generation of reactive oxygen species, which induce myocardial damage, all of which can impair cardiac function over time [1]. To mitigate the cardiovascular risk, several protective strategies have been developed and implemented. These include limiting cumulative doses of anthracyclines, using less cardiotoxic alternatives or liposomal formulations, and cotreatment with cardioprotective agents. Dexrazoxane is the only treatment that acts directly on one of the cardiotoxic mechanisms of anthracycline by inhibiting the formation of the topoisomerase complex, thereby reducing cell death. However, its use is restricted for high-risk patients due to concerns about side effects [2]. Other promising treatment strategies involve the use of established cardiovascular drugs such as neurohormonal blockers, statins, and sodium-glucose co-transporter-2 inibitors. While activation of neurohormonal pathways in response to myocardial injury is initially compensatory, prolonged activation ultimately leads to cardiac dysfunction and heart failure. Given the proven benefit of neurohormonal blockade in heart failure management, it is compelling to apply these therapies as a cardioprotective strategy in patients treated with anthracyclines. The results of the as-yet-unpublished SARAH trial, evaluating the cardioprotective effect of sacubitril/valsartan, were recently presented at the American Heart Association Scientific Sessions in November 2024. This mini review article will focus mainly on the role of neurohormonal blockade in anthracycline-related cardioprotection and position the findings of the SARAH trial within the context of current advancements in the field.

Multiple trials have proven the effects of neurohormonal blockade in improving survival, rehospitalizations, symptoms, and quality of life in heart failure. By targeting the maladaptive responses of the renin-angiotensin system (RAS), these medications reduce vasoconstriction, fluid retention, and cardiac remodeling. Early studies have demonstrated the potential of RAS inhibitors in the cardio-oncology setting. Cardinale et al. showed that troponin-guided treatment with enalapril prevented a decline in LVEF in patients receiving high-dose chemotherapy [3]. However, later studies have yielded mixed results, which reflect the challenge of translating subclinical improvements into long-term clinical benefits (Table 1).

**Table 1:** Randomized clinical trials of neurohormonal blockers in patients with cancer treated with anthracyclines and/or trastuzumab

Study	Patients (*n*)	Selection criteria	Intervention	Timing of intervention	Follow-up duration	Results
PRADA (extended follow-up)	120	Early breast cancer patients undergoing anthracycline-based chemotherapy ± trastuzumab	Candesartan ± metoprolol	Before chemotherapy	2 years	No significant LVEF difference between groups
CECCY	200	HER2-negative breast cancer patients receiving anthracycline-based chemotherapy (240 mg/m^2^)	Carvedilol	Before chemotherapy	6 months	No significant LVEF difference between the groups
Guglin et al	468	HER2-positive breast cancer patients receiving trastuzumab, stratified by anthracycline use	Lisinopril or carvedilol	Before trastuzumab	2 years	In patients receiving anthracyclines, lisinopril and carvedilol reduced cardiotoxicity as defined by LVEF
MANTICORE	94	HER2-positive breast cancer patients receiving trastuzumab	Perindopril or bisoprolol	Before trastuzumab	1 year	No effect on LV remodeling as the primary endpoint. Attenuated LVEF decline (secondary endpoint)
ICOS-ONE	273	Patients receiving anthracycline chemotherapy; troponin elevation detected	Enalapril	After troponin elevation	12 months	No significant difference in troponin elevation between groups
Cardiac CARE	175	Breast cancer and non-Hodgkin lymphoma patients with troponin elevation during anthracycline therapy	Carvedilol + candesartan	After troponin elevation	6 months	No significant difference in LVEF decline between groups
SARAH	114	Patients undergoing anthracycline chemotherapy with elevated hs-TnI (> 99th percentile)	Sacubitril/valsartan	After anthracyclines	24 weeks	GLS decline < 15% was significantly lower in the intervention group
SUCCOUR-MRI	105	Patients with > 12% reduction in GLS during anthracycline therapy	ACE inhibitors or ARBs + beta-blockers	After GLS reduction	12 months	Significantly smaller decline in LVEF in the intervention group
PROACT	111	Patients with breast cancer or non-Hodgkin lymphoma undergoing high-dose anthracycline-based chemotherapy	Enalapril or standard care without enalapril	Before chemotherapy		No significant difference in troponin T (≥ 14 ng/L) between groups

The addition of a neprilysin inhibitor to angiotensin receptor blockers has been a significant advancement in heart failure treatment. The effect is enhanced by increasing natriuretic peptides, adrenomedullin, bradykinin, and other vasoactive substances, leading to additional vasodilation, enhanced natriuresis, and reduced cardiac fibrosis. Evidence from heart failure studies has demonstrated that angiotensin receptor neprilysin inhibitors (ARNI) are superior to RAS inhibitors alone.

SARAH was a single-center, double-blind, placebo-controlled trial testing the efficacy of the ARNI sacubitril/valsartan in preventing subclinical left ventricular (LV) dysfunction in patients receiving anthracycline-based chemotherapy. Patients were randomized after chemotherapy and were only eligible for inclusion if they had evidence of cardiac injury, defined as cardiac troponin I > 99th percentile after any anthracycline infusion. A total of 114 patients were randomized to either sacubitril/valsartan or placebo, with up-titration after 2 and 4 weeks to a target dose of 97/103 mg b.i.d. Patients were stratified according to predicted cumulative dose of anthracyclines. The primary outcome was defined as a ≥ 15% reduction in global longitudinal strain (GLS) from baseline to 24 weeks.

The majority (> 80%) of patients were women treated for breast cancer with a mean age of 52 years. The trial was positive as the primary outcome occurred significantly less frequently in the ARNI group compared to the placebo group (7.1% vs. 25.0%; OR 0.23, 95% CI 0.07–0.75; *p* = 0.015). Overall, there was a 0.5% absolute GLS improvement in the ARNI group and a 1.5% decline in the placebo group, corresponding to relative changes of + 2.5% and − 7.6%, respectively (*p* = 0.001). This effect on the primary endpoint was independent of risk factors such as mean indexed cumulative dose of anthracycline, HER2 positivity, hypertension, and age. Some secondary outcomes also favored ARNI, including CMR-derived LVEF, left ventricular end-systolic volume, and echocardiographic left ventricular end-diastolic volume. Although a numerically lower proportion of patients experienced a > 10% decrease in LVEF to a final value of < 53% in the ARNI group compared to placebo (3.7% vs 11.3%), this was not statistically significant. The secondary clinical event endpoints, including symptomatic heart failure, heart failure hospitalization, and all-cause mortality, were rare (5 patients in total), and there was no significant difference between the groups. The safety profile of ARNI was generally favorable, with comparable rates of adverse effects to placebo (19.3% vs. 15.8%; *p* = 0.806). However, hypotension occurred more frequently in the ARNI group (14.0% vs. 1.8%; *p* = 0.032).

Findings from the SARAH trial are important contributions to the expanding field of cardio-oncology. Several trials have yielded mixed results in the efficacy of prevention of cardiotoxicity by neurohormonal blockade. In general, two approaches have been used: the broad administration of preventive therapy and the targeted approach, where treatment is initiated based on predefined markers of cardiotoxicity, such as elevated cardiac troponin, or cardiac dysfunction, such as GLS or LVEF.

Early studies demonstrated that elevation in cardiac troponin I was strongly related to future cardiotoxicity [4, 5]. This has inspired trials with a “troponin-triggered approach.” The ICOS-ONE trial randomized patients receiving anthracyclines to either preventive treatment with enalapril or starting enalapril after troponin elevation [6]. The trial was not placebo-controlled, and the incidence of cardiotoxicity was very low at 1.1% overall, thereby lacking statistical power to provide robust answers. Similarly, in the Cardiac Care trial, treatment with carvedilol and candesartan was initiated in patients with troponin I elevations [7]. However, there was no association between troponin I levels and changes in LVEF. This was recently supported by another study that found no association between troponin I or T and future LVEF decline [8]. These mixed results raise several important questions, including whether troponins are reliable predictors for future clinical cardiotoxicity.

The SARAH trial offers a promising perspective that troponin elevation can still serve as a valuable biomarker to identify patients at risk for developing cardiotoxicity. A cardioprotective effect may be more evident in a higher-risk cohort receiving higher doses of anthracyclines, as cardiotoxicity is believed to be dose-dependent. In the SARAH trial, the majority of patients received lower anthracycline doses (*n* = 94 < 300 mg/m^2^, *n* = 20 > 300 mg/m^2^). This likely contributes to the lower incidence of cardiotoxicity and may limit the ability of the trial to assess the benefit of the cardioprotective intervention tested.

The SARAH trial used the change in GLS as its primary endpoint, reflecting on its value as a marker of early myocardial dysfunction. In a meta-analysis, GLS predicted future LVEF decline in patients receiving anthracycline therapy [9]. The initial SUCCOUR trial evaluated patients randomized to either GLS-guided or LVEF-guided cardioprotection strategies, showing no significant difference in change of LVEF at 3 years [10]. Addressing the limitations of the study, the recent SUCCOUR-MRI trial randomized patients with a decline of 12% in GLS, but preserved LVEF, to either usual care or treatment with RAS inhibitors and beta-blockers [11]. The primary endpoint, mean LVEF on MRI after 12 months, was significantly better in the intervention group compared to the usual care group. Although these findings suggest that interventions targeting early changes in GLS can preserve LVEF over time, other studies, such as the earlier PRADA trial, did not demonstrate a significant difference in LVEF at the 2-year follow-up despite improved GLS in candesartan-treated patients [12]. Notably, the design of the two trials differed; SUCCOUR-MRI used a decline in GLS to trigger treatment in patients with early cardiotoxicity, while PRADA assessed GLS as an outcome of treatment in a primary prevention setting. These design differences may potentially explain the varying results.

For most patients in the SARAH trial, treatment with ARNI was initiated *after* completion of chemotherapy, possibly indicating that myocardial damage had already occurred. This raises the question of whether starting therapy earlier can yield a greater benefit. This will be addressed by the ongoing PRADA 2 trial, which randomizes a comparable patient population to ARNI vs placebo from the *beginning* of chemotherapy [13]. Moreover, the relatively short observation time in the SARAH trial limits conclusions about the long-term clinical benefits, and follow-up studies are eagerly anticipated.

In conclusion, the SARAH trial provides valuable insights into the role of ARNI in the prevention of subclinical cardiac dysfunction in patients undergoing anthracycline therapy. The findings align with a growing recognition in the field of cardio-oncology that a targeted approach may be more efficient than broad administration in an unselected population. This is supported by several recent trials which highlight the difficulties in achieving meaningful protection in groups with a low incidence of cardiotoxicity-related events. Furthermore, future trials evaluating other heart failure medications, including dapagliflozin, empagliflozin, and vericiguat, could provide valuable knowledge of additional cardioprotective strategies. However, one of the key challenges in the field of cardioprotection has been the surrogate endpoints used in the trials: change in GLS or LVEF, rather than CV events per se. To bridge this gap, larger trials with extended follow-up periods are necessary. An even more ambitious vision involves the development or repurposing of more targeted cardioprotective therapies, aiming for greater efficacy and fewer side effects.

## Data Availability

No datasets were generated or analyzed during the current study.
